# Stress Triaxiality in Anisotropic Metal Sheets—Definition and Experimental Acquisition for Numerical Damage Prediction

**DOI:** 10.3390/ma15113738

**Published:** 2022-05-24

**Authors:** Felix Rickhey, Seokmoo Hong

**Affiliations:** Department of Automotive and Mechanical Engineering, Kongju National University, Cheonan 31080, Korea; frrickhey@kongju.ac.kr

**Keywords:** stress triaxiality, anisotropy, sheet metal, ductile fracture, triaxiality failure diagram, digital image correlation

## Abstract

Governing void growth, stress triaxiality (*η*) is a crucial parameter in ductile damage prediction. *η* is defined as the ratio of mean stress to equivalent stress and represents loading conditions. Attempts at introducing material anisotropy in ductile damage models have started only recently, rendering necessary in-depth investigation into the role of *η* here. *η* is commonly derived via finite elemnt (FE) simulation. An alternative is presented here: based on analytical expressions, *η* is obtained directly from the strains in the critical zone. For anisotropic materials, *η* associated with a specimen varies with yield criterion and material (anisotropy). To investigate the meaning of triaxiality for anisotropic materials, metal sheets made of dual phase steel DP780, and zirconium alloy Zirlo are chosen. Analytical expressions for *η* are derived for three popular yield criteria: von Mises, Hill48 and Barlat89. Tensile tests are performed with uniaxial tension, notch, and shear specimens, and the local principal strains, measured via digital image correlation (DIC), are converted to h. The uniaxial tension case reveals that only the anisotropic yield criteria can predict the expected *η* = 1/3. The ramifications associated with anisotropy become apparent for notched specimens, where *η* differences are highest; for shear specimens, the yield criterion and material-dependence is relatively moderate. This necessitates *η* and, consequently, the triaxiality failure diagram (TFD) being accompanied by the underlying yield criterion and anisotropy parameters. As the TFD becomes difficult to interpret, it seems more advantageous to provide pairs of principal strain ratio *β* and failure strain. Suggestions for deriving representative *β* and *η* are made.

## 1. Introduction

For the numerical prediction of damage in a sheet metal structure during forming or in crash events, a plethora of macroscopic ductile damage models has been developed. To reduce weight and consumption of resources, and thus increase efficiency, material of often-low ductility is exploited up to close to their strain limit, which renders these models increasingly important. Despite the large variety of damage models available today, what they share is the incorporation of the strong influence of triaxiality *η* on damage and fracture. This insight can be traced back to the early works by McClintock [1,2], Rice and Tracey [3], Hancock and Mackenzie [4], and Beremin [5], who already pointed out the importance of *η* in ductile damage. The inclusion of the anisotropic yield in damage models has gained attention recently.

Ductile damage models can be divided into uncoupled and coupled models. Uncoupled, in this context, means that failure is judged based on the current state (e.g., equivalent strain, maximum principal stress, plastic dissipation, or some other measure) of a “virgin” material, i.e., without accounting for the damage-induced material softening with increasing deformation. Early prominent models are those by Rice and Tracey [3] and Johnson and Cook [6]; more recent models were presented, for example, by Bai and Wierzbicki [7,8], Ebnoether and Mohr [9], or Lou, Huh, and Yoon [10], who extended their work to anisotropic plasticity and damage [11]. Coupled, on the other hand, means that this material softening is considered by gradually decreasing the material’s resistance to further deformation. This penalization of the material performance can be realized through reducing Young’s modulus, the stress tensor, or some other measure, in accordance with the amount and direction of deformation, deformation history, loading conditions, etc.

From a microstructure perspective, ductile damage occurs in the sequence of void nucleation–void growth–void coalescence (moderate to high triaxialities) or by shear band formation (low triaxialities). Early approaches were presented by McClintock [1,2]. This damage mechanism has been the foundation of the early physics-based model by Gurson [12], which was refined by Tvergaard and Needleman [13,14,15]. The resulting Gurson–Tvergaard–Needleman (GTN) model has, since then, undergone numerous modifications and extensions, with different foci among them, including anisotropy [16,17]. 

Continuum mechanics-based phenomenological models (CDM models) whose thermodynamic foundations were laid by Kachanov in 1958 [18], Lemaitre [19] and Chaboche [20], have been more successful in this regard [21,22,23]. Badreddine and Saanouni [24] extended the approach to plastic and damage anisotropies. What CDM-based models have in common is (i) that a damage driver is defined, and (ii) once a damage driver threshold is surpassed, damage accumulates depending on the damage driver increment, weighted by triaxiality and other parameters. To overcome the weakness of unsatisfactory damage prediction under non-monotonic loading conditions, the coupled GISSMO (generalized incremental stress state dependent damage model) was developed based on the uncoupled Johnson–Cook model [25]. The formulation relies on the triaxiality failure diagram (TFD), which provides the change of critical and failure strains with triaxiality. Damage initiates once a (remaining) formability parameter surpasses a threshold, and failure occurs once a Lemaitre-type damage parameter reaches another threshold. Both parameters are functions of *η*.

This shows that, regardless of damage model, the reliability of damage prediction stands and falls with the accuracy of *η*. *η* is defined as mean (or hydrostatic) stress normalized by equivalent stress. It is, thus, a function of the first stress invariant (*I*_1_) and the second deviatoric stress invariant (*J*_2_) and, as such, represents the plastic constraint. In the low *η* region, shear fracture dominates, whereas at higher *η*, void growth governs damage; a transition zone separates them [26]. The Lode angle, another factor, becomes relevant in the low triaxiality region, e.g., [27,28,29], although it was reported by Badreddine et al. [30] that this explicit Lode angle sensitivity vanishes once plastic anisotropy is coupled with damage anisotropy.

Numerical simulations on different scales have underscored the significance of *η* regarding void evolution [31,32,33]. Recent studies have directed their focus toward materials with low ductility: advanced steel [34], Al alloys [26,35], and Mg alloys [36,37]. Since the evolution of the fracture strain with *η* is complex, specimens of various shapes have been designed to investigate the damage behavior for a large range of *η*, i.e., loading conditions [38]: shear [39,40], biaxial tension [41,42], mixed tension-shear [43], and compression [44]. Recently, tests under non-proportional loading paths, with *η* jumps [45] or continuously changing *η* [46,47], have also drawn interest, as they offer insight into the fracture behavior under complex loading conditions.

To facilitate modelling and reduce computational costs, sheet material is often simplified by assuming isotropic yielding [9,26,32,48,49], although this is rarely the case and is bound to lead to inaccuracies. For isotropic metal sheets, von Mises yielding is second to none, making *η* uniquely associated with a specimen shape and independent of the material. For anisotropic materials, however, the choice of the yield model is not unambiguous and, thus, is the expression for and value of *η*, which vary with yield model and material. As, consequently, the abscissa (*η*-position) of a point extracted for a given specimen is not fixed, the TFD becomes difficult to interpret. A different approach was presented by Jia and Bai [50], who formulated their eMMC (strain-based modified Mohr–Coulomb) model in a purely strain space, thereby evading any strain-to-stress conversion.

The role of anisotropic yielding in damage has gained attention only recently. Park et al. [51] modified the isotropic Lou–Huh damage model by substituting the Hill48 yield condition, to account for the anisotropic evolution of the strain towards fracture, but for the mildly anisotropic DP980. A similar attempt was made by Lou and Yoon [52] and applied to an Al alloy. Earlier, the same authors [53] used linear transformation to develop an anisotropic fracture criterion that accounts for the dependence of fracture on the orientation with regard to the rolling direction. Li et al. [54] combined a Lemaitre-based damage criterion with the Barlat89 yield criterion and applied the resulting model to a moderately anisotropic Al alloy. The GISSMO was further generalized to MAGD (named after its LS-Dyna keyword *Mat_Add_Generalized_Damage), where the orientation-dependence (rolling, transverse, and diagonal directions) and other damage drivers can be accounted for, which allows for high flexibility. Andrade et al. [55] investigated the influence of the yield criterion, but they limited anisotropy to the use of the normal anisotropy and to the Hill48 yield criterion; in addition, the material was only mildly anisotropic. Bhadauria et al. [56] noted the change of triaxiality with the mean r-value for the Hill48 yield criterion. 

**Figure 1:**
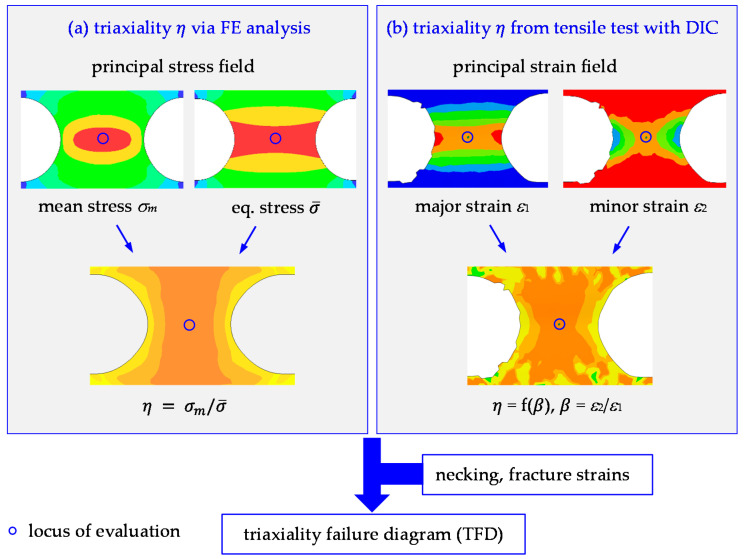
Evaluation of triaxiality *η* from FE analysis (**a**) vs. directly from tensile test using DIC (**b**). The FE approach is based on computed mean stress *σ_m_* and equivalent stress $\bar{\sigma }$, whereas the experimental approach relies on minor-to-major strain ratio *β* (=*ε*_2_/*ε*_1_) data in the central specimen region where failure occurs (blue circles). The computed triaxialities are subsequently used, along with corresponding necking and fracture strains, to establish the TFD, the basis for damage models such as GISSMO or MAGD. The principle is illustrated for a notched specimen (N5).

This shows that the role of *η*, as the ductile damage-driving parameter of isotropic as well as anisotropic sheets, is uncontested. However, research on the determination of *η*, as well as a clear understanding of the yield model and anisotropy-dependence, is lacking. Depending on how *η* is defined, it may assume quite different values, and this needs clarification and quantification. In this paper, the role of the yield model and the meaning of *η* for anisotropic materials, in general, are investigated by an analytical-experimental approach. Microstructural features are not accounted for specifically, but they are merged into the anisotropic flow curves and r-values of the continuum model. Basically, there are two ways of obtaining *η*-values, as outlined in Figure 1: (i) the test is simulated by FE analysis, and from the resulting stress tensor, *η* = *σ_m_*/$\bar{\sigma }$ is calculated, where *σ_m_* and $\bar{\sigma }$ are mean stress and equivalent stress, respectively; (ii) the strain full field for a certain test is obtained, and *η* is calculated from the minor strain increment (d*ε*_2_)-to-major strain increment (d*ε*_1_) ratio *β*^′^. While method (i), the conventional routine, requires yield and damage models that can accurately reproduce the actual strain field, method (ii) requires an analytical solution, based on the chosen yield criterion, to convert principal strain ratio *β* to *η*. In either case, however, stresses cannot be measured directly, so the accuracy of the obtained triaxiality value depends on the appropriateness of the yield condition.

To avoid the additional step of numerical simulation, which is bound to introduce further uncertainty, approach (ii) is applied here. Analytical expressions are derived for the von Mises, Hill48, and Baralat89 yield models that allow the conversion of *β* to *η*. Two sheet metals are chosen that vastly differ in their degree of anisotropy. Tensile tests with nine different uniaxial tensions, as well as notched and shear specimens, were carried out. Strains in the critical deformation zone were measured via digital image correlation (DIC) and converted to *η* using the analytical expressions. The evolutions of the principal strain ratios and triaxialities are analyzed, and the differences are discussed. Finally, a method for deriving a representative *η*, based on a representative *β*, for a certain specimen is suggested. The *η*, so obtained, can then be used to establish the TFD (together with critical and fracture strains), the backbone of damage models such as GISSMO or MAGD.

## 2. Theory—Yield Model-Dependent Analytical Solutions for Triaxiality

In this section, analytical expressions allowing the conversion of principal strains to triaxiality are derived. This is done by transforming the stress-based definition of *η* (=hydrostatic stress/equivalent stress) into a principal strain-based expression via the flow rule. This procedure is applied, here, to the von Mises, Hill48, and Barlat89 yield criteria but it may be applied to any other yield criterion, such as Yld2000, although it may be difficult to derive closed-form expressions. The resulting equations are used in Section 4 to analyze the triaxiality evolution under different loading conditions. It is to be noted that, commonly, the *J*_2_-based von Mises equivalent stress is taken to normalize the hydrostatic stress. However, for highly anisotropic materials, it may assume values that do not represent the actual stress state, and the equivalent stress associated with the applied yield criterion (which is used here) is considered more meaningful.

Before deriving these analytical expressions, we introduce the principal stress ratio *α*, which is defined as

(1) $$\alpha \equiv \frac{\sigma_{2}}{\sigma_{1}}$$

where *σ*_1_ and *σ*_2_ are major and minor in-plane principal stresses, respectively. |*σ*_1_| ≥ |*σ*_2_| restricts *α* to [−1, 1]. Further, principal strain ratio *β* and instantaneous principal strain ratio (in rate form) *β*^′^ are defined as

(2) $$\beta \equiv \frac{\varepsilon_{2}}{\varepsilon_{1}}\;\;\;;\;\;\;\beta^{\prime }\equiv \frac{{\dot{\varepsilon }}_{1}}{{\dot{\varepsilon }}_{2}}$$

where *ε*_1_ and *ε*_2_ (${\dot{\varepsilon }}_{1}$ and ${\dot{\varepsilon }}_{2}$) are the major and minor in-plane principal strains (strain rates), respectively. In this study, tests are carried out so that, at least up to necking (ultimate tensile stress, UTS), *β* = *β*^′^, provided r-values remain constant. While *β* is used to find a representative *η*-value that can be input to numerical damage models, *β*^′^ is used to show the evolution of *η* in the high strain region.

*η* is defined as the ratio of mean stress *σ_m_* to equivalent stress $\bar{\sigma }$. Assuming plane stress conditions (*σ*_3_ = 0) leads to

(3) $$\eta =\frac{\sigma_{m}}{\bar{\sigma }}=\frac{\sigma_{1}+\sigma_{2}}{3\bar{\sigma }}$$

For the expression for $\bar{\sigma }$, we choose three oft-applied yield models: (i) the isotropic von Mises yield criterion; (ii) the anisotropic Hill48 yield criterion; (iii) the anisotropic Barlat89 yield criterion. Only final expressions are presented here; their derivation can be found in Appendix A.

### 2.1. Von Mises Yield Criterion

For the von Mises yield criterion, we arrive at

(4) $$\eta =\frac{1+\alpha }{3\sqrt{1-\alpha +\alpha^{2}}}\mathrm{sign}\left(\sigma_{1}\right)=\frac{1+\beta^{\prime }}{\sqrt{3}\sqrt{1+\beta^{\prime }+{\beta^{\prime }}^{2}}}\mathrm{sign}\left(\sigma_{1}\right)$$

where the sign term, sign(*σ*_1_) = *σ*_1_/|*σ*_1_|, is included to cover the compressive region.

### 2.2. Hill48 Yield Criterion

For the Hill48 yield criterion, *η* becomes

(5) $$\eta =\frac{1+\alpha }{3\;\sqrt{1-\frac{2r_{0}}{1+r_{0}}\alpha +\frac{r_{0}\left(1+r_{90}\right)}{r_{90}\left(1+r_{0}\right)}\alpha^{2}}}\mathrm{sign}\left(\sigma_{1}\right)\;\;;\;\;\alpha =\frac{\beta^{\prime }\frac{1+r_{0}}{r_{0}}+1}{\frac{1+r_{90}}{r_{90}}+\beta^{\prime }}$$

where *r*_0_ and *r*_90_ are the r-values from uniaxial tension tests with sheet specimens, whose axes are parallel and transverse to the rolling direction (*θ* = 0° and 90° orientations), respectively.

### 2.3. Barlat89 Yield Criterion

For the Barlat89 yield criterion [57] *η* can be expressed as

(6) $$\begin{array}{l} \eta \left(\alpha \right)=\frac{2^{1/m}}{3}\frac{1+\alpha }{{\left[a{\left(1+\left|h\alpha \right|\right)}^{m}+\left(2-a\right){\left|1-h\alpha \right|}^{m}\right]}^{1/m}}\mathrm{sign}\left(\sigma_{1}\right)\;\;\;;\\ \beta^{\prime }\left(\alpha \right)=h\;\;\frac{{\left|h\alpha \right|}^{m-1}\mathrm{sign}\left(\sigma_{1}\right)-\frac{2-a}{a}{\left|1-h\alpha \right|}^{m-1}}{1+\frac{2-a}{a}{\left|1-h\alpha \right|}^{m-1}} \end{array}$$

where *a* = 2 − *c*, and *h* are material coefficients directly related to the r-values. The exponent *m* depends on the microstructure; we use *m* = 8 for DP780, as is suggested for FCC materials and *m* = 3 for Zirlo. The value for Zirlo is assumed based on the exponent of the Cazacu–Barlat yield model suitable for hcp materials [58]. (The assumption of *m* = 3 for Zirlo was validated through FE analysis: tensile tests with shear and notch specimens were simulated, and *β* evolutions compared; the results obtained with *m* = 3 were in good agreement with experimental data.) In principal stress space, the shear term, involving *p*, vanishes. Note that an implicit method is required to obtain *η* from *β*^′^.

While, for unixial tension (UT), *η* is, according to theory, always 1/3 for the yield criteria considered here, Equations (5) and (6) demonstrate that *η* values not only depend on the loading conditions but also on the yield criterion and *r_θ_* (and *m* for Barlat89). Due to the *r_θ_*-dependence of *η* in Equations (5) and (6), we expect deviations from the isotropic case will grow with anisotropy. For loading along a locally constant strain path, *β* can be replaced by *β*^′^.

## 3. Experiment—Tensile Tests with DIC

To analyze the evolution of *η* under different loading conditions, tensile tests were conducted with specimens of different shapes and principal strains measured locally in the specimen center. The strains were then converted to triaxiality using the equations presented in Section 2.

**Figure 2:**
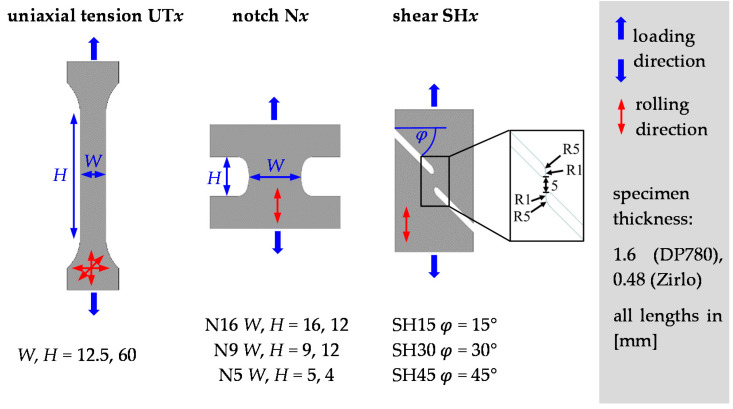
Geometries of the selected uniaxial tension (UT0, UT45, UT90), notch (N5, N9, N16), and shear (SH15, SH30, SH45) specimens. By stretching the specimens in the loading direction, different loading conditions (different principal stress and strain ratios) are induced in the center region, which is the area where fracture occurs. Every specimen type is represented by a unique point in the TFD.

All tests were carried out on the universal testing machine (UTM) Shimadzu AG-X (Shimadzu Corporation, Kyoto, Japan) with a load capacity of 50 kN. The sheet specimens shown in Figure 2 were designed to induce different loading conditions in the non-negative *η*-region, at least for isotropic sheets: uniaxial tensile specimens (UT) with orientations *θ* = 0°, 45°, and 90°; three shear specimens with slit angels of 15°, 30°, and 45° (drawn with the loading direction), SH15, SH30, and SH45, respectively; three notched specimens with minimum bridge widths of 5, 9, and 16 mm (N5, N9, N16). For isotropic sheets, *η* in the center region was found to be approximately 0.08 (SH15), 0.05 (SH30), 0.0 (SH45), 0.57 (N5), 0.49 (N9), and 0.52 (N16).

Two materials were chosen to investigate the problem: (1) the dual phase steel DP780, with martensite islands embedded in a ferrite matrix (0.12% C, 0.5% Si, 1.8% Mn, 0.35% Cr; P, S, Si < 0.01%), is used in automotive production for safety-relevant parts and shows mild anisotropy; (2) the zirconium alloy Zirlo (zirconium low oxidation; 1.0% Sn, 1.0% Nb, 0.1% Fe), used for nuclear spacer grids, shows pronounced anisotropy. The sheet thicknesses are 0.48 (Zirlo) and 1.6 mm. Considering the specimen dimensions, plane stress conditions are applicable.

**Figure 3:**
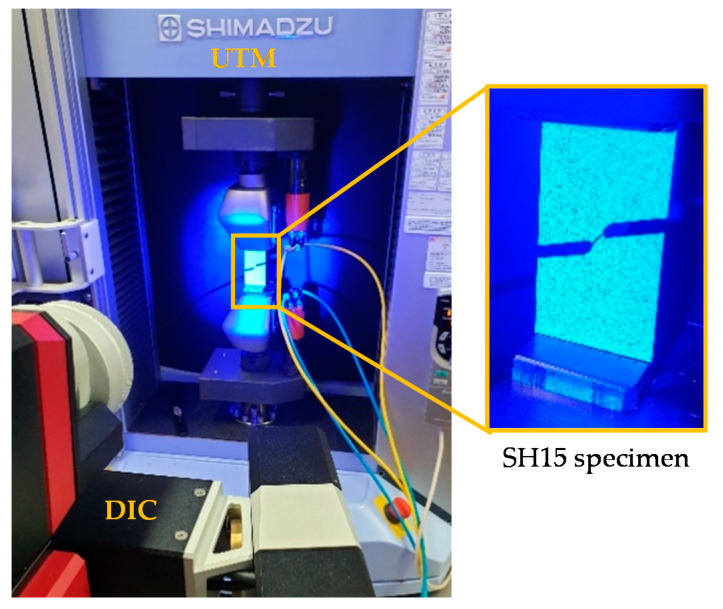
Tensile test setup with the DIC system. The enlarged image shows a fractured Zirlo SH15 specimen after the test was halted.

**Figure 4:**
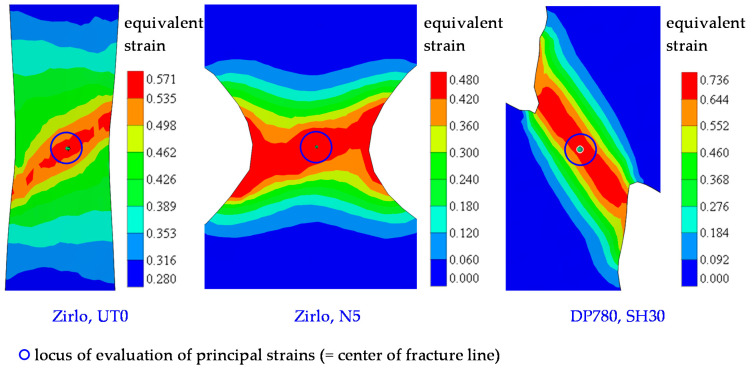
Acquisition of principal strains, in the center of the critical zone, by digital image correlation (DIC). The examples show uniaxial tension (here, Zirlo UT0; **left**), notch (here, Zirlo N5; **center**), and shear specimens (here, DP780 SH30; **right**). The distribution plots give the von Mises equivalent strain shortly before fracture.

The tensile test setup is shown in Figure 3. The test velocity was set to 5 mm/min for the UT specimens and 3 mm/min for the others, due to their lower displacement to failure. Digital image correlation (DIC) was employed to determine the strain in the specimen center where fracture commences (Figure 4). To do so, a speckled pattern was sprayed onto the specimen surface; the irregular pattern was then recorded by a high-speed camera; details are given in Table 1. For DP780, the frame rate was set to 1 Hz and full resolution of 2752 × 2200 [px] was used. For Zirlo, we started with a frame rate of 1 Hz (resolution 2752 × 2200 [px]) and switched to higher frame rates of 25 Hz (2752 × 2200) and 44 Hz (1376 × 1100, in binning mode) for the last 200 and 400 images, respectively, to get a detailed view of the high strain region. The images were then converted to displacement and strain fields using GOM Aramis Professional 2018. Each test was performed four times for DP780, twice with 25 Hz, and twice with 100 Hz for Zirlo. Due to the different frame rates and resolutions used for Zirlo, the measured maximum strains may be different. Resolution, frame rate (in conjunction with external displacement rate), facet size, and point distance are known to play an important role when it comes to absolute strain values [59]. However, apart from the very last frames before fracture, this does not apply to strain (increment) ratios.

## 4. Results

First, the UT*x* test data from Section 3 were used to determine the sheet metals’ r-values, *r_θ_*. The influence of the r-values on the *η*-*β* dependence for the two materials is briefly presented. We then investigate the evolutions of *β* and *β*^′^ and, applying the analytical expressions in Section 2, of *η* and *η*^′^ for each of the three yield criteria and the different specimens.

The procedure for determining *η* experimentally is as follows: Major and minor principal strain increments are computed for the first frame in Aramis Profesional 2019. The principal strain increment ratio *β*^′^ is calculated. From *β*^′^, *α* is determined for each yield criterion and then plugged into the respective expressions for *η* Equations (4)–(6). This procedure is repeated for all subsequent frames until fracture occurs. That the von Mises yield criterion cannot accurately predict the *β*^′^-evolution in highly anisotropic materials is obvious, yet it is used, here, to demonstrate the consequences the assumption of an isotropic material can have. As the UT*x* case is special, insofar as *η* = 1/3, it gives valuable first insight into the appropriateness of the yield criterion and its parameters. The other two cases (N*x* and SH*x*) are, then, to demonstrate the influence of the yield criterion on the resulting *η*-values for the two materials.

### 4.1. Yield Criterion-Dependent η-Relations for DP780 and Zirlo

**Figure 5:**
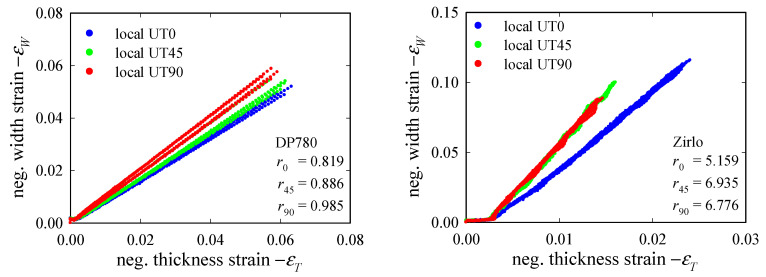
Plots of negative width strain (–*ε_W_*) against negative thickness strain (–*ε_T_*), up to the maximum load *F*_max_, for UT0, UT45, and UT90 specimens. The r-values *r*_0_, *r*_45_, and *r*_90_ are taken as the average slopes of the DP780 (**left**) and Zirlo (**right**). The low strain region, where elastic strains are significant, is not considered in the evaluation or *r_θ_*. The results show good repeatability, and the slopes remain constant, providing a reliable evaluation of *r_θ_*. All experimental data (4 data sets per specimen type) are plotted in one color per specimen type.

The force-displacement data from the three UT*x* tests were converted to true stress-strain data, assuming isotropic elasticity with Young’s moduli *E* = 190 and 82.3 GPa and Poisson’s ratios *n* = 0.33 and 0.37 for DP780 and Zirlo, respectively. The simplification of the elastic region, by Hooke’s law, is justified by the circumstances that it is the moderate-to-high strain region that is of interest for damage modeling. First, r-values (or Lankford coefficients) are determined for the orientations *θ* = 0°, 45°, and 90°. r-values are understood, here, in an instantaneous sense, i.e., width-to-thickness strain increment ratio *r_θ_* = d*ε_W_* /d*ε_T_*, and they were derived from local strains in the specimen center (Figure 4, UT*x*). Thickness strains were calculated assuming volume conservation under plastic deformation. Apart from the very small (elastic) strain region, the slopes of the (–*ε_W_*)-(–*ε_T_*) curves in Figure 5 are approximately constant up to the maximum load. The average slopes were, therefore, taken as (constant) r-values, although some deviation from the initally constant values in the post-critical region was previously reported by the authors [60]: (*r*_0_, *r*_45_, *r*_90_; $\bar{r}$) = (0.819, 0.886, 0.985; 0.894) and (5.159, 6.935, 6.776; 6.429) for DP780 and Zirlo, respectively, where $\bar{r}$ denotes normal anisotropy. The very high r-values obtained for the Zirlo sheet indicate very low thinning.

**Figure 6:**
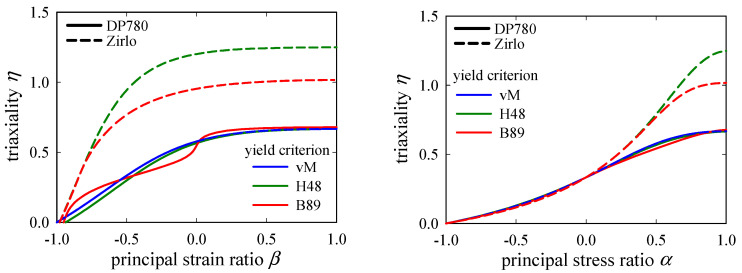
Theoretical change of triaxiality *η* with principal strain and stress ratios: *β* (**left**) and *α* (**right**), respectively, according to von Mises (vM), Hill48 (H48), and Barlat89 (B89) yield criteria Equations (4)–(6), respectively. The plots demonstrate that, while the influence of the yield criterion is small for the mildly anisotropic DP780, it can become significant for the highly anisotropic Zirlo sheet metal. Due to symmetry with respect to the horizontal axis, only the region *σ*_1_ ≥ 0 is shown here.

The changes of *η* with principal strain and stress ratios, *β* and *α*, according to the equations in Section 2, i.e., Equations (4)–(6), are plotted in Figure 6. The plots are restricted to non-negative *σ*_1_. While for the mildly anisotropic DP780 the three curves are quite similar, the highly anisotropic Zirlo differences between the yield criteria are significant, with the Hill48 curve being far above the von Mises curve, and the Barlat89 curve is in between. In the region *β* ≈ [–1, −0.75] or *α* ≈ [−1, 0.5], the anisotropic yield criteria provide nearly identical *η*-*β* and *η*-*α* relations, and differences become significant only for higher *β* and *α*. Further, the sensitivity of *η* to changes in *β* becomes pronounced around *η* = 0, rendering reliable evaluation of *η* in this region difficult for a material with a high degree of anisotropy and a large exponent *m*.

### 4.2. Uniaxial Tension Specimens (UTx)

**Figure 7:**
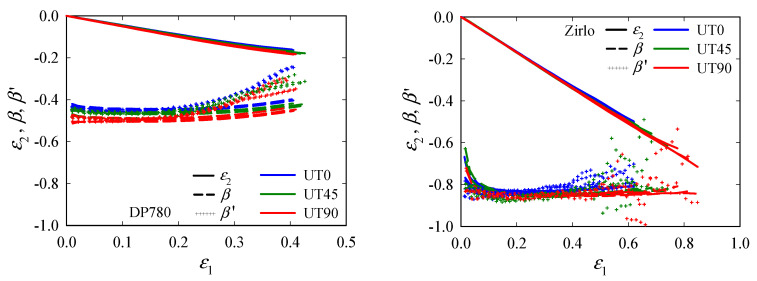
Evolutions of the minor principal strain *ε*_2_, principal strain ratio *β*, and instantaneous principal strain ratio *β*^′^, with the major principal strain *ε*_1_ in the center of uniaxial tension (UT0, UT45, and UT90) specimens; DP780 (**left**) and Zirlo (**right**). *β* and *β*^′^ were calculated from the principal strains obtained by digital image correlation (DIC). All experimental data (four data sets per specimen type) are plotted in one color per direction.

The evolutions of *ε*_2_, *β*, and *β*^′^ with *ε*_1_, are plotted in Figure 7. Independent of *θ*, the strain path is constant up to strains of *ε*_1_ ≈ 0.2 for DP780, and ≈0.4~0.5 for Zirlo. The subsequent development is expected to be influenced by the transition from a 2D to a 3D stress state, as well as damage and non-constant r-values. The departure toward plane strain (*β* = 0) is indicated, although it is not reached; this is presumed to be due to a very sudden increase that could not be captured or an insufficient resolution. *β*^′^ varies slightly with *θ*, in the order *β*^′^_UT0_ > *β*^′^_UT45_ > *β*^′^_UT90_, for both materials.

**Figure 8:**
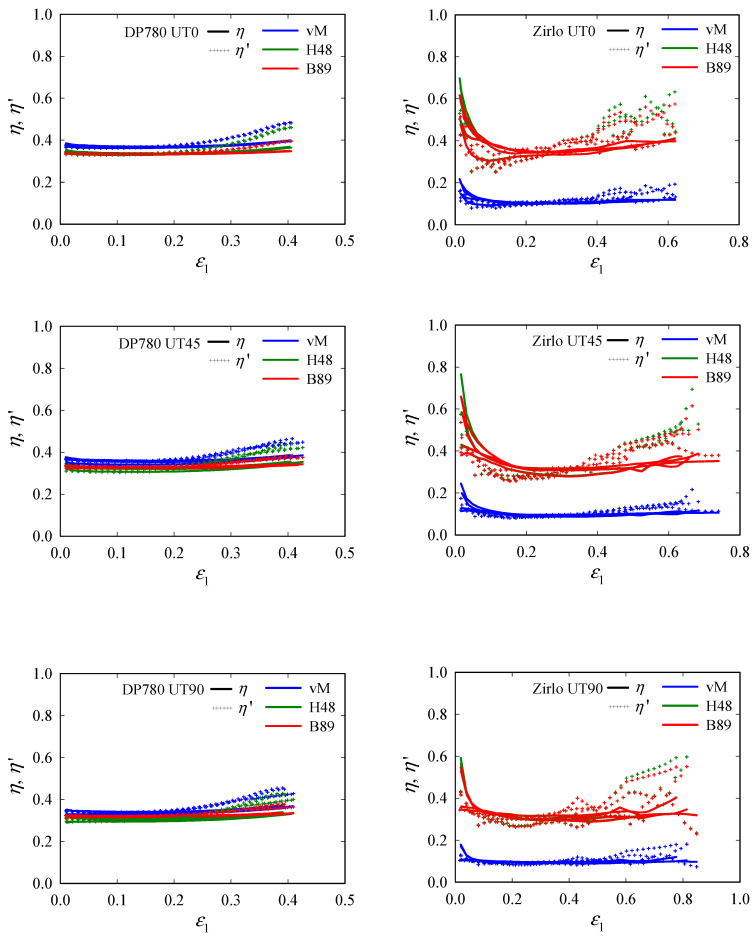
Evolutions of triaxiality *η* and instantaneous triaxiality *η*′, with major principal strain *ε*_1_ in the center of uniaxial tension (UT0, UT45, and UT90) specimens. All experimental data (four data sets per specimen type) are plotted in one color per direction: DP780 (**left**) and Zirlo (**right**). For both materials, *η*, and thus *η*′, begin to increase, at higher strains, toward fracture. While for DP780, differences between the yield criteria are rather small, they become apparent for the highly anisotropic Zirlo, where the Hill48 and Barlat89 yield models give values close to the expected value of *η* = 1/3 in the lower strain region.

The same strain data and the r-values given in Section 3 were then used to calculate *η* for the three yield criteria. The triaxiality evolutions with *ε*_1_ are plotted in Figure 8. As Figure 6 already indicated, the influences of yield criterion and anisotropy are obvious. For DP780, the influence of the yield criterion is negligible, for all yield criteria provide similar values, which are very close to the theoretical value of 1/3 for uniaxial tension. For Zirlo, however, differences are pronounced: *η*-values, based on the von Mises yield criterion, are far below 1/3, highlighting its inappropriateness for materials with higher anisotropy. The Hill48 and the Barlat89 criteria, on the other hand, provide values quite close to 1/3. Further, triaxiality reaches constancy for only a relatively small *ε*_1_ range—the higher fluctuation, observed here, is due to the higher sensitivity of *η* to small changes in *β* or *β*^′^—before it starts increasing.

### 4.3. Notched Specimens (Nx)

**Figure 9:**
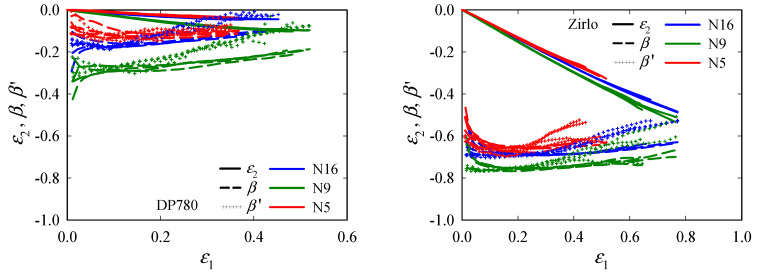
Evolutions of the minor principal strain *ε*_2_, principal strain ratio *β*, and instantaneous principal strain ratio *β*^′^, with major principal strain *ε*_1_ in the center of notched (N16, N9, N5) specimens: DP780 (**left**) and Zirlo (**right**). *β* and *β*^′^ were calculated from the principal strains obtained by digital image correlation (DIC). All experimental data (four data sets per specimen type) are plotted in one color per direction.

The same procedure was then applied to the N*x* specimens. Figure 9 provides the evolutions of *ε*_2_, *β*, and *β*^′^, with *ε*_1_ for both materials. As was the case for UT*x*, *β*^′^ increases as the fracture is approached; for DP780, *β*^′^ = 0 is nearly reached. Due to the high anisotropy, the tests with Zirlo N*x* specimens do not provide plane strain conditions and give principal strain ratios that are much different from what one would expect for notched specimens, which were initially designed to investigate the plane strain region (for isotropic materials).

**Figure 10:**
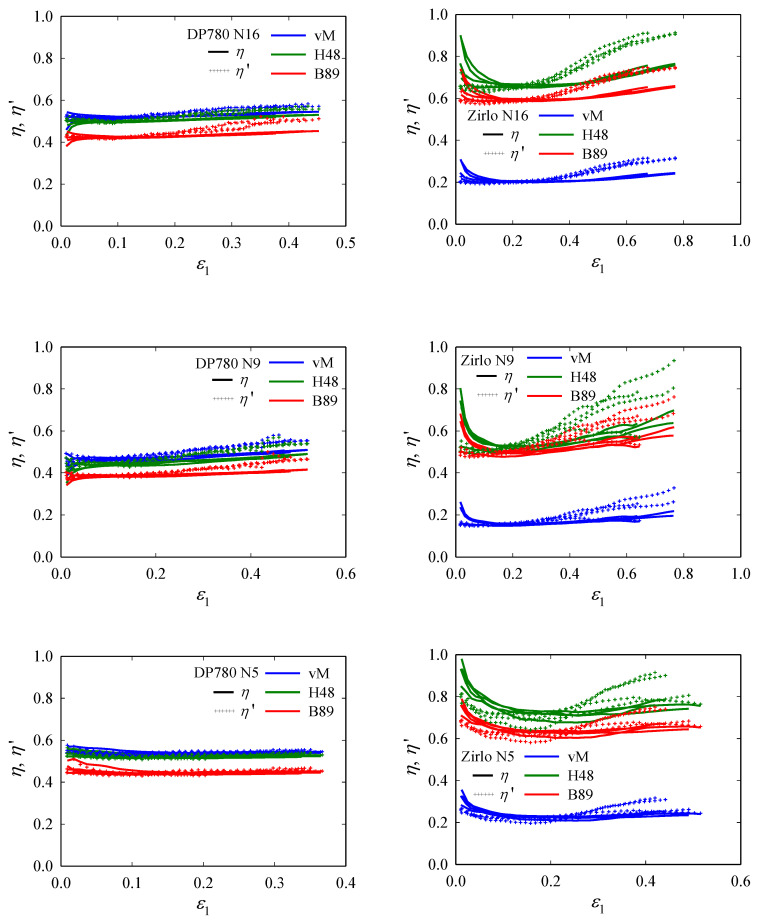
Evolutions of triaxiality *η* and instantaneous triaxiality *η*′, with major principal strain *ε*_1_ in the center of notched (N16, N9, N5) specimens. All experimental data (four data sets per specimen type) are plotted in one color per direction: DP780 (**left**) and Zirlo (**right**). For DP780, the increase in *η*′ toward fracture ranges from small (N16, N9) to nearly naught (N5), whereas for Zirlo, the increase is pronounced, with only a small strain range where it remains approximately constant. By contrast with UT*x*, the different yield criteria give quite different results for *η* and *η*′.

Resultant *η*-*ε*_1_ plots are shown in Figure 10. For the notched specimens, we find that, similar to UT*x*, the *η*-evolution in DP780 sheets shows mild to no increase up to fracture, while the Zirlo curves increase significantly with *ε*_1_, indicating that loading conditions start to change toward plane strain at relatively low *ε*_1_. By contrast with UT*x*, the differences between the two anisotropic yield criteria become obvious for N*x* specimens. For DP780, von Mises and Hill48 give similar values, which are ≈0.1 above those for Barlat89, independent of the type of notched specimen considered. For Zirlo, by contrast, all three yield criteria give different values, with the Hill48 criterion giving the highest and the von Mises criterion giving the lowest values for *η* and *η*′. Based on the Barlat89 criterion, the N*x* specimens span a range of *η* ≈ 0.45~0.52 (DP780) and ≈ 0.55~0.72 (Zirlo), with N9 and N5 giving the lowest and highest values, respectively.

### 4.4. Shear Specimens (SHx)

**Figure 11:**
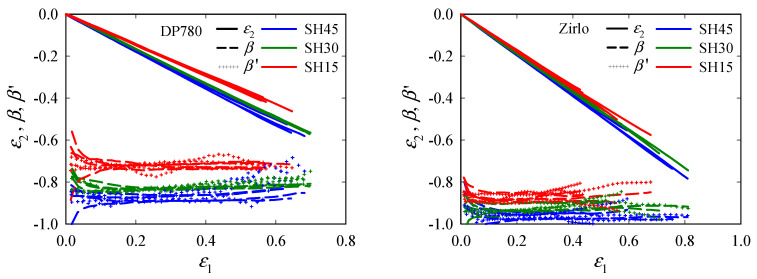
Evolutions of the minor principal strain *ε*_2_, principal strain ratio *β*, and instantaneous principal strain ratio *β*^′^, with major principal strain *ε*_1_ in the center of shear (SH45, SH30, SH15) specimens: DP780 (**left**) and Zirlo (**right**). *β* and *β*^′^ were calculated from the principal strains obtained by digital image correlation (DIC). All experimental data (four data sets per specimen type) are plotted in one color per direction.

Finally, the shear case is investigated. The evolutions of *ε*_2_, *β*, and *β*^′^, with *ε*_1_, are given in Figure 11. By contrast, with UT*x* and N*x*, *β*, *β*^′^ ≈ const. for both materials and all SH*x* specimens. *β* and *β*^′^ range between −0.7~−0.9 and −0.85~−1.0 for DP780 and Zirlo, respectively.

**Figure 12:**
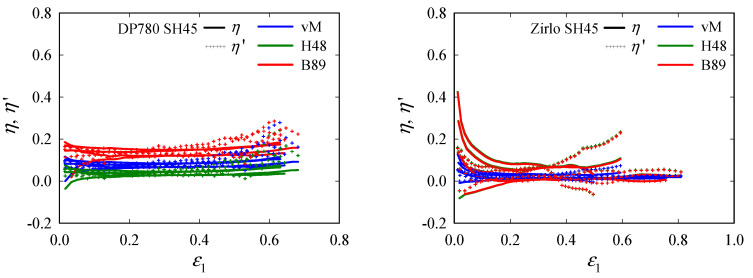

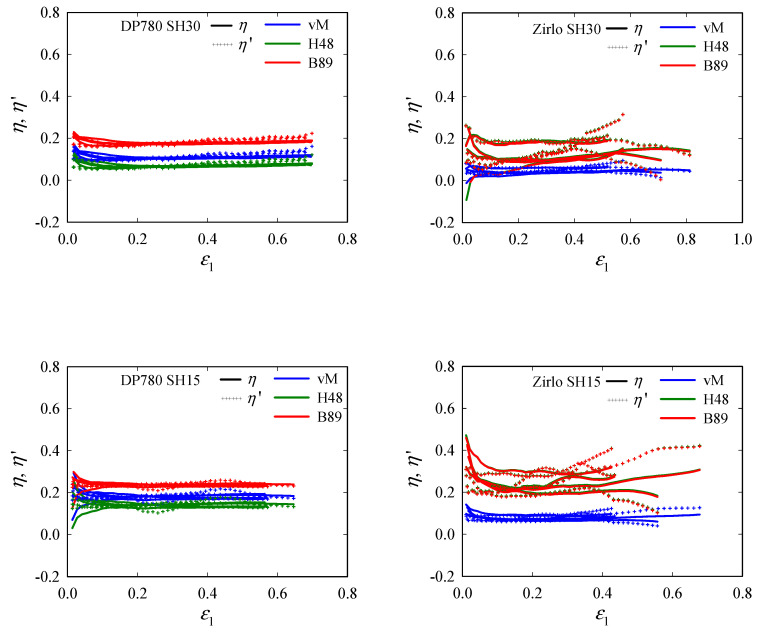
Evolutions of the triaxiality *η* and instantaneous triaxiality *η*′, with major principal strain *ε*_1_ in the center of the shear (SH45, SH30, SH15) specimens. All experimental data (four data sets per specimen type) are plotted in one color per direction: DP780 (**left**) and Zirlo (**right**). For both materials, *η* and *η*′ can be considered to remain approximately constant with increasing *ε*_1_, despite the rather high fluctuation for Zirlo, due to the sensitivity of *η* and *η*′ to small changes in *β* and *β*^′^, respectively. The different yield criteria yield quite different *η* and *η*′.

The triaxiality evolutions are depicted in Figure 12. As expected from *β*^′^, the triaxiality remains more or less constant. The fluctuation observed for Zirlo, for some samples, is attributed to the steep slope d*η*/d*β*, close to *β* = −1 for the anisotropic yield criteria, as mentioned before. Again, the influence of the yield criterion is more pronounced for Zirlo. For DP780, the Barlat89 criterion yields somewhat higher *η* and *η*′ than the other two, which give similar results. For Zirlo, on the other hand, while differences are quite small for SH45, they become pronounced for SH15, where Hill48 and Barlat89 produce *η* and *η*′ much higher than von Mises.

## 5. Discussion

For nine specimen geometries, *η* evolutions were derived directly from the DIC strain full field, using analytical expressions for three yield criteria. In light of the fact that the goal, here, is not to find the most appropriate yield model for the materials under different loading conditions but to demonstrate the influence anisotropy can have when it comes to triaxiality-based damage prediction, the following statements can be made:

1. The choice of the yield criterion can have a strong influence on the resulting triaxiality for anisotropic materials. This influence varies further with the degree of anisotropy (magnitude of and differences between r-values) and exponent *m*.
2. The UT*x* case shows the risks inherent to the assumption of material isotropy: for the highly anisotropic Zirlo, the von Mises criterion gives triaxialities of ≈0.1, which is well below the theoretical value of 1/3 in the pre-critical region. Both anisotropic yield criteria, however, give values close to 1/3.
3. While for the mildly anisotropic DP780, the von Mises criterion gives triaxialities comparable to Hill48, for the highly anisotropic Zirlo, differences are pronounced. Based on the von Mises criterion, the *η* range for the specimen geometries used in this study does not exceed [0, 0.2].
4. While small for UT*x* and SH*x* specimens, differences between the Hill48 and Barlat89 yield models become significant for N*x* specimens.
5. Due to the high sensitivity in the *β* = −1 region, the analysis of the shear region can be challenging, depending on the yield criterion and exponent *m*.
6. The material dependence of *η* dilutes the advantages of the TFD for anisotropic materials, i.e., the unique relationship between the *η*-value and a specimen type. It is more practical to provide *β* (or *β*^′^), as it is independent of yield criterion or anisotropy and can be measured directly.

**Figure 13:**
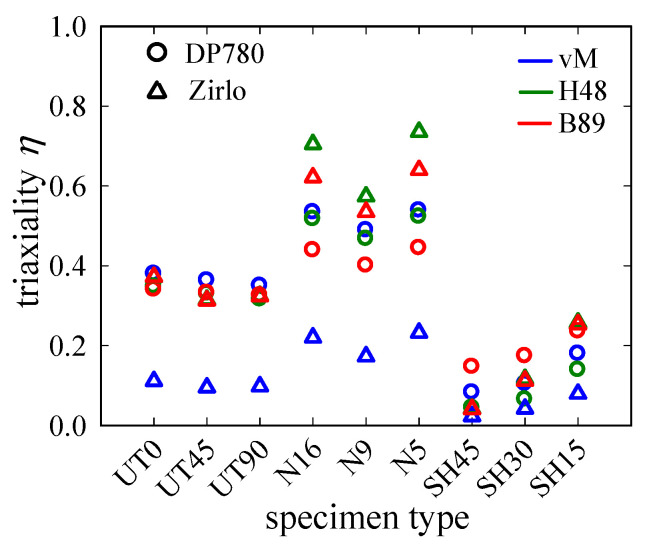
Influence of the yield criterion and material on triaxiality, calculated from the representative principal strain ratio for DP780 and Zirlo. This is a graphical representation of the values in Table 2 and Table 3. The influence becomes pronounced for N*x* specimens and the highly anisotropic Zirlo.

Direct extraction of *η* from experimental (strain) data has the advantage that the evaluated *η* is directly associated with the critical and fracture strains, which facilitates straight experimental investigation of fracture behavior. Damage does not compromise results as long as plane stress conditions remain valid. Apart from SH*x* specimens for both materials and N5 specimens for DP780, *η* increases from an initially more or less constant value with ongoing deformation, signifying a change in the stress state due to damage initiation or a change in r-values. A representative value that is then used for the TFD, and thus numerical damage modeling, needs to be determined. Due to the sensitivity of *η* to *β* for certain loading conditions, it seems advantageous to first determine a representative *β* and then calculate the corresponding *η*-value. Taking the average slope of the *ε*_2_-*ε*_1_ curve as representative *β*, we arrive at the triaxialities in Table 2 and Table 3 for DP780 and Zirlo, respectively. The yield criterion influence is plotted in Figure 13, where the large discrepancy for N*x* becomes apparent.

## 6. Summary and Concluding Remarks

In this study, we investigated, for two sheet metals with highly different degrees of anisotropy, the influence of the yield criterion and anisotropy on triaxiality *η*, which is a crucial parameter in ductile damage. The study is meant to shed light on the meaning of triaxiality for anisotropic materials. It was shown that *η* can be directly obtained from the DIC strain full field; the required analytical expressions were provided for three yield criteria. Doing so, we make sure that *η* can be correctly assigned to the corresponding fracture strains. Further, this bypasses any FE simulation, which introduces further uncertainty. It was demonstrated that *η* becomes strongly yield criterion-dependent, especially for the highly anisotropic sheet metal Zirlo. As the *η*-value associated with a specimen shape is not universal, it, as well as the TFD, should be accompanied by the yield criterion and material properties. It, therefore, seems to be advantageous to provide the principal strain ratio *β* instead to eliminate this dependence; *β* can then be converted to *η* based on a yield criterion. In addition, this allows purely experimental analysis of the fracture behavior, independent of anisotropic yield. A method for deriving a representative *η*-value from the average slope of the *ε*_2_-*ε*_1_ curve was presented. Together, with the corresponding critical and fracture strains, this value constitutes the TFD, the backbone of damage models such as the MAGD.

## Figures and Tables

**Table 1:** DIC settings for tensile tests.

DIC System	GOM Aramis 3D Camera 6M System
full resolution	6 Mpx: 2752 × 2200 [px]
used resolution	DP780: full resolution (1 Hz) Zirlo: full resolution (25 Hz), 1376 × 1100 (44 Hz, in binning mode)
measuring volume	length×width×depth = 150 × 120 × 105 [mm]
image resolution	0.055 mm/px
camera angle	25°
facet size/point distance	13 px/8 px
strain measuring accuracy	0.01%

**Table 2:** Representative *β*- and *η*-values for DP780. The *η*-values are calculated by inserting the representative principal strain ratio (*β*) into the respective expressions, Equations (4)–(6).

Specimen	Repr. *β*	Repr. *η* (vM)	Repr. *η* (H48)	Repr. *η* (B89)
UT0	−0.428	0.380	0.348	0.340
UT45	−0.454	0.363	0.331	0.332
UT90	−0.475	0.350	0.316	0.326
N16	−0.128	0.534	0.517	0.439
N9	−0.232	0.489	0.468	0.400
N5	−0.115	0.539	0.523	0.445
SH45	−0.865	0.083	0.044	0.147
SH30	−0.832	0.105	0.065	0.174
SH15	−0.722	0.179	0.140	0.236

**Table 3:** Representative *β*- and *η*-values for Zirlo. The *η*-values are calculated by inserting the representative principal strain ratio (*β*) into the respective expressions, Equations (4)–(6).

Specimen	Repr. *β*	Repr. *η* (vM)	Repr. *η* (H48)	Repr. *η* (B89)
UT0	−0.822	0.111	0.372	0.373
UT45	−0.846	0.095	0.315	0.313
UT90	−0.842	0.098	0.324	0.324
N16	−0.664	0.220	0.705	0.621
N9	−0.731	0.173	0.574	0.535
N5	−0.647	0.232	0.736	0.640
SH45	−0.961	0.023	0.043	0.041
SH30	−0.929	0.042	0.116	0.112
SH15	−0.870	0.080	0.258	0.254

## Data Availability

Not applicable.

## Appendix A

The derivation of the principal strain ratio-based expressions for triaxiality *η* are presented for the von Mises, Hill48, and Barlat89 yield criteria. Results for the von Mises yield criterion can be found elsewhere, but they are added here for consistency. The formulae are given in terms of principal strain rate ratio *β*′; however, under continuous loading along a constant strain path, *β* = *β*′. Plane stress conditions (thickness stress *σ*_3_ = 0) are assumed.

### Appendix A.1. Von Mises Yield Criterion

For general plane stress conditions, the von Mises equivalent stress $\bar{\sigma }$ $\bar{\sigma }$ can be expressed as

(A1) $$\bar{\sigma }=\sqrt{\sigma_{x}^{2}+\sigma_{y}^{2}-\sigma_{x}\sigma_{y}+3\sigma_{xy}{}^{2}}$$

where *σ_x_*, *σ_y_* and *σ_xy_* are the three components of the stress tensor **σ**, expressed as a 3 component vector **σ** = (*σ_x_* *σ_y_* *σ_xy_*)^T^. Equation (A1) can be rewritten in matrix form as

(A2) $$\bar{\sigma }=\sqrt{\left(R\sigma \right)\cdot \sigma }$$

where

(A3) $$R=\frac{1}{2}\left[\begin{array}{ccc} 2 & -1 & 0\\ -1 & 2 & 0\\ 0 & 0 & 6 \end{array}\right]$$

Elastic strains are neglected. The associated flow rule provides

(A4) $$d\varepsilon /d\lambda =\partial \bar{\sigma }/\partial \sigma$$

where, equivalent to **σ**, **ε** is the strain tensor expressed as a 3-component vector **ε** = (*ε_x_* *ε_y_* 2*ε_xy_*)^T^ and *dλ* is the plastic multiplier. Inserting Equation (A2) into Equation (A4) gives

(A5) $$d\varepsilon /d\lambda =\frac{\partial \sqrt{\left(R\sigma \right)\cdot \sigma }}{\partial \sigma }=\frac{1}{2\bar{\sigma }}\frac{\partial \left(R\sigma \right)\cdot \sigma }{\partial \sigma }=\frac{1}{2\bar{\sigma }}\frac{\partial \sigma^{T}R\sigma }{\partial \sigma }=\frac{R\sigma }{\bar{\sigma }}$$

Considering work conjugation ($\bar{\sigma }d\bar{\varepsilon }=\sigma d\varepsilon$), we get

(A6) $$d\bar{\varepsilon }=\frac{1}{\bar{\sigma }}\sigma \cdot d\varepsilon \;\;\;\to \;\;\;\frac{d\bar{\varepsilon }}{d\lambda }=\frac{1}{\bar{\sigma }}\sigma \cdot \frac{\partial \bar{\sigma }}{\partial \sigma }=\frac{1}{{\bar{\sigma }}^{2}}\sigma \cdot \left(R\sigma \right)=1$$

which means that the plastic multipier is the equivalent strain.

The expression for *η* is obtained as follows. In principal stress space, Equation (A1) can be expressed in terms of principal stress ratio *α* = *σ*_2_/*σ*_1_ as follows

(A7) $$\bar{\sigma }=\sqrt{\sigma_{1}^{2}+\sigma_{2}^{2}-\sigma_{1}\sigma_{2}}=\left|\sigma_{1}\right|\sqrt{1-\alpha +\alpha^{2}}$$

where *σ*_1_ and *σ*_2_ are major principal stress and minor principal stress, respectively. Plugging Equation (A7) into the expression for *η* yields

(A8) $$\eta =\frac{\sigma_{m}}{\bar{\sigma }}=\frac{\sigma_{1}\left(1+\alpha \right)/3}{\left|\sigma_{1}\right|\sqrt{1-\alpha +\alpha^{2}}}=\frac{1+\alpha }{3\sqrt{1-\alpha +\alpha^{2}}}\mathrm{sign}\left(\sigma_{1}\right)$$

In terms of the principal stress and strain rate ratios, the relation becomes

(A9) $$\beta^{\prime }=\frac{d\varepsilon_{2}}{d\varepsilon_{1}}=\frac{\partial \bar{\sigma }/\partial \sigma_{2}}{\partial \bar{\sigma }/\partial \sigma_{1}}=\frac{2\sigma_{2}-\sigma_{1}}{2\sigma_{1}-\sigma_{2}}=\frac{2\alpha -1}{2-\alpha }$$

Plugging Equation (A9) into Equation (A8) eventually gives the desired expression:

(A10) $$\eta =\frac{1+\beta^{\prime }}{\sqrt{3}\sqrt{1+\beta^{\prime }+{\beta^{\prime }}^{2}}}\mathrm{sign}\left(\sigma_{1}\right)$$

### Appendix A.2. Hill48 Yield Criterion

For general plane stress conditions, the Hill48 yield criterion can be written as

(A11) $$\bar{\sigma }=\sqrt{\sigma_{x}{}^{2}-\frac{2r_{0}}{1+r_{0}}\sigma_{x}\sigma_{y}+\frac{r_{0}\left(1+r_{90}\right)}{r_{90}\left(1+r_{0}\right)}\sigma_{y}{}^{2}+\frac{r_{0}+r_{90}}{1+r_{0}}\frac{2r_{45}+1}{r_{90}}\sigma_{xy}{}^{2}}$$

or, in matrix form,

(A12) $$\bar{\sigma }=\sqrt{\left(S\sigma \right)\cdot \sigma }$$

where **S** is a matrix of *r_θ_*-dependent components, which can be determined by comparison with Equation (A11):

(A13) $$S=\left[\begin{array}{ccc} 1 & \frac{2r_{0}}{1+r_{0}} & 0\\ \frac{2r_{0}}{1+r_{0}} & \frac{r_{0}}{r_{90}}\frac{1+r_{90}}{1+r_{0}} & 0\\ 0 & 0 & \frac{r_{0}+r_{90}}{1+r_{0}}\frac{2r_{45}+1}{r_{90}} \end{array}\right]$$

Since the Hill48 criterion can be expressed in the same form as the von Mises criterion above, application of the flow rule and work conjugation must lead to the same result, i.e.,

(A14) $$\frac{d\bar{\varepsilon }}{d\lambda }=1$$

(the expressions for $\overset{-}{\varepsilon }$, however, vary with yield criterion).

Albeit more tedious mathematically, for finding an expression for *η*, the same procedure used for the von Mises criterion can be applied to the other yield criteria. For the Hill48 criterion, the equivalent stress in principal stress space becomes

(A15) $$\bar{\sigma }=\left|\sigma_{1}\right|\sqrt{1-\frac{2r_{0}}{1+r_{0}}\alpha +\frac{r_{0}\left(1+r_{90}\right)}{r_{90}\left(1+r_{0}\right)}\alpha^{2}}$$

With Equation (A15), the triaxiality becomes

(A16) $$\eta =\frac{1+\alpha }{3\;\sqrt{1-\frac{2r_{0}}{1+r_{0}}\alpha +\frac{r_{0}\left(1+r_{90}\right)}{r_{90}\left(1+r_{0}\right)}\alpha^{2}}}\mathrm{sign}\left(\sigma_{1}\right)$$

Making use of the flow rule again, we can write for the principal strain rate ratio

(A17) $$\beta^{\prime }=\frac{d\varepsilon_{2}}{d\varepsilon_{1}}=\frac{\frac{r_{0}\left(1+r_{90}\right)}{r_{90}\left(1+r_{0}\right)}\alpha -\frac{r_{0}}{1+r_{0}}}{1-\frac{r_{0}}{1+r_{0}}\alpha }=\frac{\frac{1+r_{90}}{r_{90}}\alpha -1}{\frac{1+r_{0}}{r_{0}}-\alpha }$$

Solving for *α* and plugging into Equation (A16) yields the desired expression.

### Appendix A.3. Barlat89 Yield Criterion

For the Barlat89 yield criterion, a plastic potential Φ is defined as

(A18) $$\begin{array}{l} \Phi =a{\left|K_{1}+K_{2}\right|}^{m}+a{\left|K_{1}-K_{2}\right|}^{m}+c{\left|K_{2}\right|}^{m}=2{\bar{\sigma }}^{m}\\ \;\;\;\;\;K_{1}=\frac{\sigma_{x}+h\sigma_{y}}{2}\;\;\;;\;\;\;K_{2}=\sqrt{{\left(\frac{\sigma_{x}-h\sigma_{y}}{2}\right)}^{2}+p^{2}\sigma_{xy}^{2}}\\ \;\;\;\;\;a=2-c=2-2\sqrt{\frac{r_{0}}{1+r_{0}}\frac{r_{90}}{1+r_{90}}}\;\;\;;\;\;\;h=\sqrt{\frac{r_{0}}{1+r_{0}}\frac{1+r_{90}}{r_{90}}}\;\;\;\\ \;\;\;\;\;m>0,\;\in \mathbb{N}\;;\;a,\;p,\;h>0\;;\;a<2 \end{array}$$

where *K*_1_ and *K*_2_ denote stress invariants. *a* = 2 − *c*, *h*, *p* are material coefficients related to *r_θ_*, and *m* depends on the crystal structure. Again, we start by deriving the plastic multiplier, now for the Barlat89 yield criterion. Together with the flow rule, we arrive at

(A19) $$\frac{d\bar{\varepsilon }}{d\lambda }=\frac{1}{\bar{\sigma }}\sigma \cdot \frac{\partial \bar{\sigma }}{\partial \sigma }=\frac{1}{m\Phi }\sigma \frac{\partial \Phi }{\partial \sigma }$$

The partial derivative becomes

(A20) $$\frac{\partial \Phi }{\partial \sigma }=\frac{m}{2}\left(\begin{array}{c} a\frac{{\left|K_{1}+K_{2}\right|}^{m}}{K_{1}+K_{2}}\left(1+\frac{\sigma_{x}-h\sigma_{y}}{2K_{2}}\right)+a\frac{{\left|K_{1}-K_{2}\right|}^{m}}{K_{1}-K_{2}}\left(1-\frac{\sigma_{x}-h\sigma_{y}}{2K_{2}}\right)+c\frac{{\left|K_{2}\right|}^{m}}{K_{2}}\frac{\sigma_{x}-h\sigma_{y}}{2K_{2}}\\ ha\frac{{\left|K_{1}+K_{2}\right|}^{m}}{K_{1}+K_{2}}\left(1-\frac{\sigma_{x}-h\sigma_{y}}{2K_{2}}\right)+ha\frac{{\left|K_{1}-K_{2}\right|}^{m}}{K_{1}-K_{2}}\left(1+\frac{\sigma_{x}-h\sigma_{y}}{2K_{2}}\right)-hc\frac{{\left|K_{2}\right|}^{m}}{K_{2}}\frac{\sigma_{x}-h\sigma_{y}}{2K_{2}}\\ \frac{2p^{2}\sigma_{xy}}{K_{2}}\left[a\frac{{\left|K_{1}+K_{2}\right|}^{m}}{K_{1}+K_{2}}-a\frac{{\left|K_{1}-K_{2}\right|}^{m}}{K_{1}-K_{2}}+c\frac{{\left|K_{2}\right|}^{m}}{K_{2}}\right] \end{array}\right)$$

Inserting Equation (A20) into Equation (A19), we find

(A21) $$\begin{array}{l} \frac{d\bar{\varepsilon }}{d\lambda }=\frac{1}{2\Phi }\left[\begin{array}{l} \sigma_{x}\left(a\frac{{\left|K_{1}+K_{2}\right|}^{m}}{K_{1}+K_{2}}\left(1+\frac{\sigma_{x}-h\sigma_{y}}{2K_{2}}\right)+a\frac{{\left|K_{1}-K_{2}\right|}^{m}}{K_{1}-K_{2}}\left(1-\frac{\sigma_{x}-h\sigma_{y}}{2K_{2}}\right)+c\frac{{\left|K_{2}\right|}^{m}}{K_{2}}\frac{\sigma_{x}-h\sigma_{y}}{2K_{2}}\right)\\ +\sigma_{y}\left(ha\frac{{\left|K_{1}+K_{2}\right|}^{m}}{K_{1}+K_{2}}\left(1-\frac{\sigma_{x}-h\sigma_{y}}{2K_{2}}\right)+ha\frac{{\left|K_{1}-K_{2}\right|}^{m}}{K_{1}-K_{2}}\left(1+\frac{\sigma_{x}-h\sigma_{y}}{2K_{2}}\right)-hc\frac{{\left|K_{2}\right|}^{m}}{K_{2}}\frac{\sigma_{x}-h\sigma_{y}}{2K_{2}}\right)\\ +\sigma_{xy}\frac{2p^{2}\sigma_{xy}}{K_{2}}\left[a\frac{{\left|K_{1}+K_{2}\right|}^{m}}{K_{1}+K_{2}}-a\frac{{\left|K_{1}-K_{2}\right|}^{m}}{K_{1}-K_{2}}+c\frac{{\left|K_{2}\right|}^{m}}{K_{2}}\right] \end{array}\right]\\ \;=\frac{1}{2\Phi }\left[\begin{array}{l} \left[\sigma_{x}+h\sigma_{y}+\frac{2}{K_{2}}\left(\frac{{\left(\sigma_{x}-h\sigma_{y}\right)}^{2}}{4}+p^{2}\sigma_{xy}{}^{2}\right)\right]a\frac{{\left|K_{1}+K_{2}\right|}^{m}}{K_{1}+K_{2}}\\ +\left[\sigma_{x}+h\sigma_{y}-\frac{2}{K_{2}}\left(\frac{{\left(\sigma_{x}-h\sigma_{y}\right)}^{2}}{4}-p^{2}\sigma_{xy}{}^{2}\right)\right]a\frac{{\left|K_{1}-K_{2}\right|}^{m}}{K_{1}-K_{2}}\\ +\frac{2}{K_{2}}\left(\frac{{\left(\sigma_{x}-h\sigma_{y}\right)}^{2}}{4}+p^{2}\sigma_{xy}{}^{2}\right)c\frac{{\left|K_{2}\right|}^{m}}{K_{2}} \end{array}\right]=\frac{1}{2\Phi }\left[\begin{array}{l} 2\left(K_{1}+K_{2}\right)a\frac{{\left|K_{1}+K_{2}\right|}^{m}}{K_{1}+K_{2}}\\ +2\left(K_{1}-K_{2}\right)a\frac{{\left|K_{1}-K_{2}\right|}^{m}}{K_{1}-K_{2}}\\ +2K_{2}c\frac{{\left|K_{2}\right|}^{m}}{K_{2}} \end{array}\right]\\ \;=\frac{1}{\Phi }\left[a{\left|K_{1}+K_{2}\right|}^{m}+a{\left|K_{1}-K_{2}\right|}^{m}+c{\left|K_{2}\right|}^{m}\right]=1 \end{array}$$

Hence, as for the other two yield criteria, the plastic multiplier is the equivalent strain.

To find an expression for *η*, we switch to the principal stress space; the yield stress becomes the Hosford yield stress enriched by *h*. We arrive at

(A22) $$\eta \left(\alpha \right)=\frac{2^{1/m}}{3}\frac{1+\alpha }{{\left[a{\left(1+\left|h\alpha \right|\right)}^{m}+\left(2-a\right){\left|1-h\alpha \right|}^{m}\right]}^{1/m}}\mathrm{sign}\left(\sigma_{1}\right)$$

and the flow rule provides

(A23) $$\beta^{\prime }\left(\alpha \right)=h\;\;\frac{{\left|h\alpha \right|}^{m-1}\mathrm{sign}\left(\sigma_{1}\right)-\frac{2-a}{a}{\left|1-h\alpha \right|}^{m-1}}{1+\frac{2-a}{a}{\left|1-h\alpha \right|}^{m-1}}$$

*η* can then be calculated from *β*′ by implicitly solving for *α* and inserting this value into Equation (A22).
